# Influence of Xylanase Inclusion on Productive Performance, Egg Quality and Intestinal Health of Commercial Laying Hens Fed Energy-Reduced Diets

**DOI:** 10.3390/ani15213190

**Published:** 2025-11-02

**Authors:** Giovana Longhini, Rasha Qudsieh, Mário Lopes, Isabela Silva, Vitor Pais, Raimundo Netto, Melany Lovon, Carlos Granghelli, Douglas Faria, Lucio Araujo, Cristiane Araujo

**Affiliations:** 1 Department of Animal Science, School of Animal Science and Food Engineering, University of São Paulo, Pirassununga 13635-900, SP, Brazilvitorsouza@usp.br (V.P.); rnetto@usp.br (R.N.); mfernandezl@usp.br (M.L.); carlosgranghelli@usp.br (C.G.); defaria@usp.br (D.F.); lfaraujo@usp.br (L.A.); 2 Novus International, Inc., Chesterfield, MO 63005, USA; 3 Department of Animal Production and Nutrition, School of Veterinary Medicine and Animal Science, University of São Paulo, Pirassununga 13635-900, SP, Brazil; crsilva@usp.br

**Keywords:** bacterial diversity, egg quality, histology, intestinal permeability, volatile fatty acids

## Abstract

**Simple Summary:**

In this study, we evaluated the effects of xylanase addition to wheat–soybean meal-based diets with reduced metabolizable energy (ME) on growth performance, egg quality, intestinal health and microbiota composition in laying hens. A total of 280 Lohmann LSL white laying hens were monitored from 20 to 40 weeks of age. The results showed that xylanase inclusion at 50 and 100 g/MT improved egg production, egg mass and shell quality, without interfering with feed intake or feed conversion. Additionally, intestinal permeability was significantly reduced, and positive changes were observed in the gut microbiota, increasing the abundance and diversity of beneficial bacterial groups. These findings suggest that dietary xylanase supplementation may serve as a valuable nutritional strategy to enhance both productivity and intestinal health in laying hens, especially when using energy-reduced diets.

**Abstract:**

This study evaluated the inclusion of increasing levels of xylanase in reduced-energy diets for commercial laying hens. A total of 280 Lohmann LSL white laying hens were equally allocated one of five dietary treatments, with seven replicates of eight hens each being a positive control: a wheat and soybean meal-based diet (PC, ME 2725 kcal/kg), a negative control diet (NC, PC minus 100 kcal) and three diets with increasing xylanase levels of 50, 100 and 150 g/MT (NC + XM50, NC + XM100 and NC + XM150, respectively). The hens were monitored from 20 to 40 weeks of age to assess productive performance, egg quality and intestinal health, including histomorphometry, permeability and microbiota composition. Xylanase inclusion at 50 and 100 g/MT significantly improved egg production and egg mass, as well as shell strength and thickness, while maintaining feed intake and feed conversion efficiency, while xylanase inclusion at 150 g/MT decreased egg production and egg mass. Additionally, intestinal permeability was significantly reduced, and positive changes were observed in the gut microbiota. Higher doses of xylanase (100 and 150 g/MT) increased bacterial abundance and diversity, with a greater presence of beneficial phyla such as *Bacteroidota*, which play an important role in gut health. There was also a reduction in *Actinobacteriota*, indicating a lower presence of potential pathogens. Changes in *Campylobacterota*, *Cyanobacteria* and *Proteobacteria* were observed, especially with the highest xylanase dose. These findings suggest that xylanase can improve laying hen performance and promote intestinal integrity and microbial balance when included in energy-reduced diets, offering a promising strategy to enhance health and productivity in commercial egg production systems.

## 1. Introduction

Poultry nutrition plays a fundamental role in productive efficiency and animal welfare. The diets commonly used in poultry farming are composed mainly of soybean meal, corn and wheat, ingredients that are highly digestible but also contain antinutritional factors that can compromise nutrient absorption. In this context, the inclusion of exogenous enzymes, such as xylanases, has proven to be an effective strategy for optimizing nutrient utilization and improving bird performance.

The dietary addition of xylanases favors digestion and nutrient absorption by stimulating the growth and activity of beneficial microorganisms in the digestive tract. This enzymatic action enhances nutrient metabolizability, thereby supporting improved bird performance and reducing energy expenditure in tissue renewal and muscle growth, in addition to contributing to better intestinal morphometry [1].

Xylanase primarily functions by reducing digesta viscosity, accelerating gastric emptying and decreasing intestinal fermentation [2]. This inhibits the growth of anaerobic microorganisms, reducing the incidence of diseases and the demand for immune cells in the intestine, which has a positive impact on bird performance [2]. This effect highlights the role of carbohydrases in increasing the availability of nutrients for muscle synthesis, avoiding the need to mobilize amino acids for energy generation [3]. Xylanase can act by specifically degrading the cell wall of arabinoxylans, which cannot be degraded by the endogenous enzymes of birds [4]. Therefore, the access to encapsulated nutrients after degradation can occur, leading to better nutrient uptake by the intestinal epithelial cells and increasing their digestibility [5]. Since more energy can be utilized from the diet with xylanase inclusion, the inclusion of high-energy ingredients in diets, such as corn, could be reduced, allowing for lower production costs [6].

Despite carbohydrases being commonly used in commercial farms, contrasts in the literature regarding its use are still present, such as concerning its effects on eggshell quality and internal egg quality, as well as a lack of information about the characterization and richness of the gut microbiota in laying hens.

Therefore, the present study aims to evaluate the inclusion of different concentrations of xylanases in diets with reduced metabolizable energy, based on corn, soybean meal and wheat bran on the productive performance, egg quality, intestinal volatile fatty acids profile and concentration, intestinal integrity and microbiota profile of commercial laying hens.

## 2. Materials and Methods

### 2.1. Experimental Design and Diet Composition

The study was conducted in the experimental shed of the Department of Animal Science of the School of Animal Science and Food Engineering (FZEA) of the University of São Paulo (USP), located at the Fernando Costa campus in Pirassununga, São Paulo. All experimental procedures were approved and reviewed by the Ethics Committee on Animal Use of FZEA/USP (protocol no. 5325130423).

A total of 280 Lohmann LSL white laying hens, 20 weeks of age, were housed in conventional cages and allocated in a completely randomized design, with five treatments and seven replicates of eight birds each. The experimental period lasted from 20 to 40 weeks of age, divided into five cycles of 28 days each. The experimental treatments are described in Table 1. The diets were corn and soybean meal-based, formulated to meet the nutritional requirements of commercial laying hens [7]. A full overview of the diet composition is presented in Table 2. All diets were provided in mash form, and feed and water were provided ad libitum throughout the experiment. A 16 h light program was maintained, following the management guidelines for Lohmann LSL hens [8]. Temperature and relative humidity parameters inside the shed were recorded daily, as well as mortality rates. The shed operates on mechanical ventilation, with the temperature set to 25 ± 1 °C during the experimental period. The feed additive used in this study was a thermostable xylanase (XM) produced by *Pichia pastoris*, which is an endoxylanase belonging to the GH11 family (CIBENZA Maxx, Novus International, Inc., Chesterfield, MO, USA), designed to improve the degradation of soluble and insoluble xylans, maximize energy utilization and improve the overall digestibility of nutrients in the diet. The product has at least 150,000 XU/g of xylanase activity. Experimental diets consisted of a positive control (PC), a commercial standard corn, soybean meal and wheat bran-based diet (2725 kcal/kg of metabolizable energy), a negative control (NC)—the positive control diet with 100 kcal reduction (2625 kcal/kg of metabolizable energy) and the NC + XM50, NC + XM100 and NC + XM150 diets had a further addition of xylanase at concentrations of 50, 100 and 150 g/MT, respectively, based on the commercial recommendations of the enzyme (50–100 g/MT) as well as inclusion rates aiming at higher XM activity in the gut.

### 2.2. Hen Performance and Egg Quality

Variables of hen performance were evaluated per replicate at the end of each 28-day cycle. From this, the following characteristics were determined: feed intake (average feed consumption per cage, g/hen/day), egg production (total number of eggs laid/number of hens per cage, expressed as a percentage of eggs/hen/day), egg mass (average egg weight multiplied by egg production percentage per cage, expressed in g/hen/day), feed conversion per dozen eggs (total feed intake/dozen eggs produced per cage) and feed conversion per egg mass (total feed intake/egg mass per cage, expressed in kg of feed/kg of egg). In addition, the percentage of dirty eggs, from total egg production, was calculated.

On the last day of each 28-day cycle, all eggs were collected for evaluation of their internal and external quality. Using a Digital Egg Tester (DET 6000, Nabel Co., Kyoto, Japan), eggs were individually analyzed to measure weight (g), eggshell resistance (kgf), albumen height (mm), yolk color and Haugh unit value. Shell thickness (mm) was obtained using a digital manual caliper (Absolute, Mitutoyo, Kawasaki-shi, Japan).

### 2.3. Intestinal Morphology and Integrity and Volatile Fatty Acid Production

At the end of the experimental period, one hen per replicate received 4.16 mg/kg of body weight of fluorescein isothiocyanate-dextran dissolved in water (FITC-d; 4000 Da; Sigma-Aldrich, St. Louis, MO, USA) via gavage, and after 2 h, blood was collected in order to indirectly assess intestinal integrity, using this marker to determine intestinal permeability. From the same hens, after euthanasia, approximately 3 cm of the ileum was collected to assess villus height and crypt depth intestinal morphometric evaluation.

The samples were sent for histological processing according to routine paraffin embedding techniques. Sections were obtained and stained using the Hematoxylin and Eosin technique [9]. After this procedure, digital photomicrographs were obtained using a 10× objective, using a microscope coupled to a high-resolution digital camera and image capture software. Next, the height and depth of 10 villi and 10 intestinal crypts per hen were quantified and measured using the Image J software, version 1.54k (NIH, Bethesda, MD, USA).

In addition, the cecal content of the euthanized hens was collected to evaluate volatile fatty acids (VFAs). For this analysis, approximately 0.4 g of cecal content was suspended in 1.6 mL of sterile milli-Q water. The concentrations of acetate, propionate, butyrate and lactate were determined by High Performance Liquid Chromatography (HPLC), a separation technique that allows the segregation of analytes prior to quantification, providing highly selective analyses. The samples were transferred to Eppendorf tubes and centrifuged in a microcentrifuge under standardized conditions appropriate for each sample. The supernatants obtained were analyzed for quantification of VFAs [10].

Excreta samples were collected from each replication at the end of the trial, by placing a clean tray under the cage. Fresh excreta samples were collected, and moisture content was determined, by weighing a fresh sample, oven drying at 55 °C for 72 h and then re-weighing the dried sample.

### 2.4. Hematological Analysis

At the end of experiment, blood samples were collected from the ulnar vein of one hen per treatment. Twelve milliliters of blood was collected from each hen and transferred immediately into test tubes with EDTA as an anticoagulant for the direct measurement of white blood cells.

### 2.5. Metagenomics Analysis

Microbial DNA extraction from fecal samples was performed through a PureLink^TM^ Microbiome DNA Purification Kit (Invitrogen, Thermo Fisher Scientific, Inc., Waltham, MA, USA) following the manufacturer’s instructions. The quality of DNA extracted was assessed by spectrophotometer and agarose gel electrophoresis. DNA sequence data were generated using the Illumina iSeq paired-end sequencing platform with reads of 2 × 150 bp. The library preparation was performed according to Illumina recommendations involving two PCRs, two purification steps, two agarose gels, quantification, normalization, multiplexing and library denaturation. The first PCR was performed for locus-specific amplification with primers flanking the V4 region of ~291 pb between 515 bp (5′-GTGYCAGCMGCCGCGGTAA-3′) and 806 bp (5′-GGACTACNVGGGTWTCTAAT-3′). MagBio HighPrep PCR (MagBio Genomics Inc., Gaithersburg, MD, USA) beads were used for purification, and the generated fragments were assessed by agarose gel electrophoresis. The second PCR was used to bond the barcodes of the Nextera XT V2 kit (Illumina Inc., San Diego, CA, USA), followed by additional purification, verification of the size library (bp) by an Agilent Technology Bioanalyzer and quantification using a fluorometric-based method (Qubit dsDNA HS Assay—Life Technologies, Carlsbad, CA, USA). Amplicons were pooled at equimolar ratios to perform sequencing. Microbial community sequencing analysis was performed at the School of Animal Science and Food Engineering of the University of São Paulo (FZEA/USP), Pirassununga, SP, Brazil. Sequenced data were assessed. First, demultiplexing sequence reads were pre-processed using dbcAmplicons version 0.9.0 to remove primers, adapters, and low-quality reads. Next, the unmerged forward and reverse reads were imported into R software (version 4.5.2, R Foundation, Vienna, Austria) and amplicon sequence variants (ASVs) were determined following the DADA2 analysis pipeline. The clustered sequences of prevalence and total abundance were compared using the Silva rRNA reference database. For analysis purposes, the relative abundance of the ASVs was calculated by dividing the counts of each taxon by the total number of counts for a given sample. Alpha-diversity was obtained and measured as Chao1, Simpson and Shannon. Alpha-diversity measures the diversity within a given environment with respect to its richness (number of taxonomic groups), evenness (distribution of abundances of the groups) or both. The alpha-diversity of samples can be calculated by community abundance using Chao1 (the abundance-based estimator of species richness), and the diversity index can be calculated by Shannon-index (the estimator of species richness and species evenness, with more weight on species richness) and Simpson (the estimator of species richness and species evenness, with more weight on species evenness). Analyses of microbiota diversity were performed using the Phyloseq Bioconductor package, Vegan R package, Phangorn R package and Decipher Bioconductor package.

### 2.6. Statistical Analysis

All data were tested for normality by the Shapiro–Wilk test, followed by the homogeneity of variables by the Levene test. The data on performance, egg quality, intestinal parameters, hematological analysis and VFA production obtained were analyzed using an ANOVA (analysis of variance) with the SAS statistical program v12.1 (2012, SAS Institute Inc., Cary, NC, USA). Each cage was considered an experimental unit for production and performance parameters, while each hen was considered an experimental unit for gut permeability and morphometry, blood parameters and fatty acid content. Significant means were separated using the Tukey test at a 5% significance level.

The statistical analysis of microbial diversity and taxonomic differences was carried out by collection of fecal samples, with each cage being considered an experimental unit. A comparison between alpha diversities for each group analyzed was performed through the non-parametric test of Kruskal–Wallis [11] and the post hoc Dunn’s test [12]. A *p*-value of ≤0.05 was considered significant. The statistical analysis of beta diversity was performed by PERMANOVA from the Qiime2 pipeline, using 10,000 permutations. All the figures and other statistical analysis were calculated in “R”. The alpha diversities were calculated by a “phyloseq” [13], “vegan” [14] and “microbiome” library [15]. The differences in the relative abundances of taxons between the analyzed groups were estimated with the Kruskal–Wallis test [11] and the post hoc Dunn’s test.

## 3. Results

### 3.1. Productive Performance and Egg Quality

The results showed that the use of xylanase (50 and 100 g/MT) improved egg production and mass by 6,39% and 7,07%, respectively (*p* < 0.001), when compared to the values of the negative control diets (Table 3). No statistical differences were observed among treatments (*p* > 0.05) for the characteristics of feed intake and feed conversion (kg/dz and kg/egg mass).

Egg weight was not significantly affected by xylanase dietary addition (Table 4), but eggs from the NC + XM50 group presented a lower Haugh unit value and album height compared to the PC (*p* = 0.002 and *p* = 0.003, respectively). However, it is worth noting that all treatment groups presented Haugh unit values above 72, which indicates that egg quality was excellent according to the USDA ratings [16]. Furthermore, the use of xylanase (100 g/MT) improved eggshell quality, increasing its resistance to rupture by 11.22% (*p* < 0.001) and its thickness by 5.54% (*p* < 0.05) when compared to the NC treatment.

### 3.2. Intestinal Permeability

A significant reduction in intestinal permeability was observed with the inclusion of enzymes in the diet (Table 5), with hens fed with different xylanase doses presenting a reduction in gut permeability.

### 3.3. Hematological Analysis

Regarding the hematological analysis, it was possible to observe a higher number of lymphocytes in hens fed diets with a dietary inclusion of 150 g/MT of xylanase (Table 6), with this group (NC + XM150) having the highest average number of lymphocytes (61.71%), with a significant difference (*p* < 0.05) in relation to the positive control group (PC), which had 43.14%. This result shows that xylanase inclusion promoted a more balanced activation of the adaptive immune system, without causing exacerbated inflammatory responses.

There were no effects of the use of enzymes on the percentage of eosinophils (*p* > 0.05); however, there was a decrease in the percentage of monocytes with the use of enzymes (*p* < 0.05), as hens fed with NC + XM150 diets presented lower monocyte rates than birds fed with the PC diet.

### 3.4. Intestinal Morphology and Short-Chain Fatty Acids

Regarding the analysis of intestinal morphology, it was observed that there were no effects of the treatments on the height of the villi, as well as the relationship between the height of the villi and the depth of the crypts (*p* > 0.05). However, the addition of 50 g/MT of enzymes to the diets increased (*p* < 0.05) the depth of the crypts (Table 7), which may indicate a higher rate of cell renewal of the intestinal mucosa. This response may represent a functional adaptation to the greater availability of substrates for absorption, promoted by the action of xylanase.

Short-chain fatty acids (SCFAs) are metabolites generated by the bacterial fermentation of dietary fiber (DF) in the hindgut. SCFAs are mainly composed of acetate, propionate and butyrate. They play a significant role in regulating intestinal health in birds. In the present study, there was a decrease in acetic acid and propionic acid in the cecum with the inclusion of 150 g/MT xylanase (*p* < 0.05). However, no significant difference in the concentration of butyric acid in the cecum of hens was observed between dietary treatments (Table 8).

### 3.5. Metagenomic Analysis

The analysis of microbial diversity indexes (Chao1, Shannon and Simpson) revealed statistically significant differences between treatments (Table 9). The Chao1 index, which estimates species richness (total number of species present), was significantly higher in the groups fed dietary xylanase compared to the control groups. The treatment with 100 g/MT and 150 g/MT of xylanase (NC + XM100 and NC + XM150) presented the highest estimated richness values (*p* < 0.05), indicating that enzyme addition promoted an intestinal environment more favorable to colonization by a greater variety of microorganisms.

The Shannon index, which considers both species richness and evenness, was also significantly higher in the NC + XM150 group (*p* < 0.05), demonstrating a more balanced and diverse microbial community. High values of this index are generally associated with a better functional stability of the intestinal ecosystem and a greater resilience to enteric challenges. In a complementary manner, the Simpson index, which measures dominance (with higher values indicating less dominance of a few species and, therefore, greater diversity), was also significantly higher in the NC + XM150 group (*p* < 0.05), reinforcing the benefits of xylanase in the positive modulation of the microbiota.

In contrast, the control groups (PC and NC) presented the lowest values in the three indexes evaluated, with the NC (negative control) group demonstrating the lowest diversity and intestinal microbial balance (Shannon and Simpson: *p* < 0.05).

**Figure 1:**
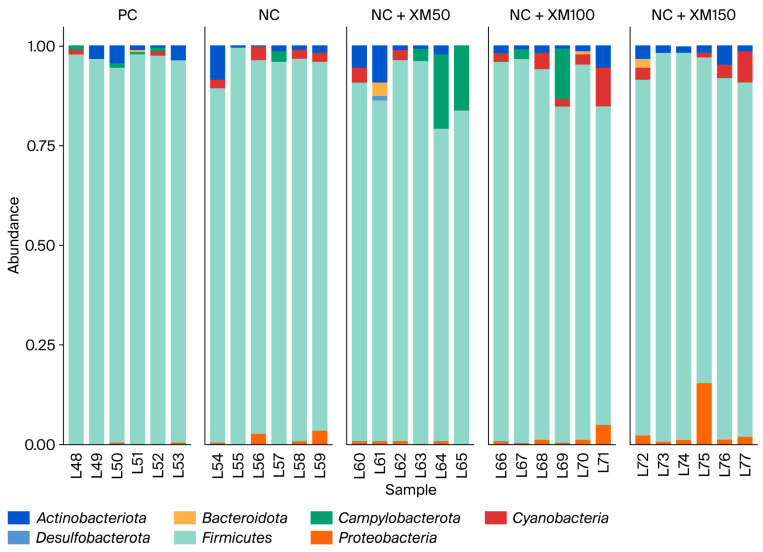
Composition of intestinal microbiota at the phylum level in laying hens subjected to increasing levels of xylanase inclusion in energy-reduced diets. PC: positive control; NC: negative control; NC + XM50: negative control + 50 g/MT xylanase; NC + XM100: negative control + 100 g/MT xylanase; NC + XM150: negative control + 150 g/MT xylanase.

**Figure 2:**
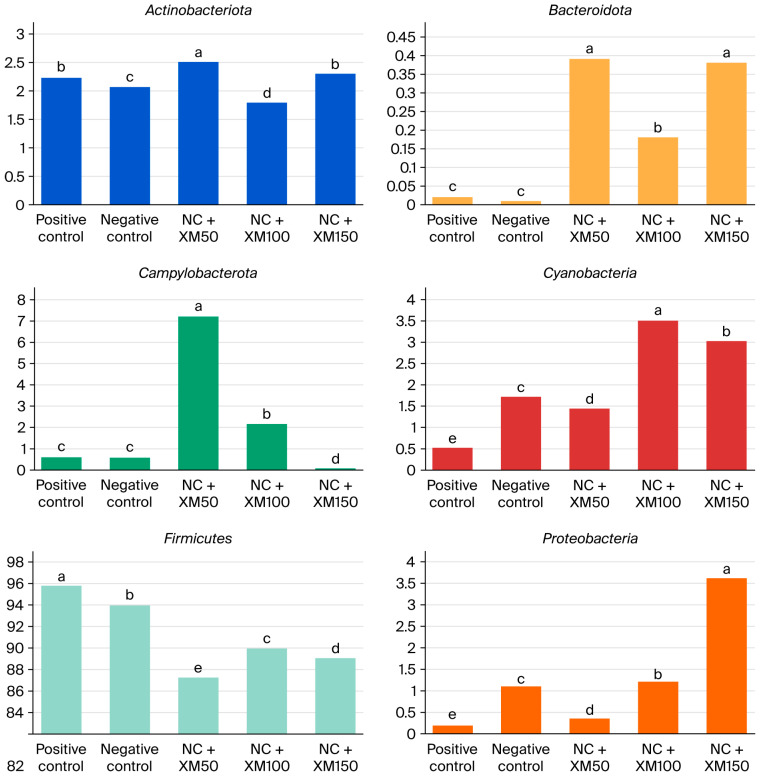
Relative abundance of the phylum *Actinobacteriota*, *Bacteriota*, *Campylobacteria*, *Cyanobacteria*, *Proteobacteria* (as a decimal fraction) and *Firmicutes* (%) in the intestinal microbiota of laying hens fed increasing levels of xylanase. NC + XM50: negative control + 50 g/MT xylanase; NC + XM100: negative control + 100 g/MT xylanase; NC + XM150: negative control + 150 g/MT xylanase. Values followed by different letters in the same graphic differ significantly by Tukey’s test (*p* < 0.05).

Taxonomic analysis showed that fecal bacterial communities were dominated by *Actinobacteriota*, *Bacteroidota*, *Campylobacterota*, *Cyanobacteria*, *Firmicutes* and *Proteobacteria* at the phylum level. The most abundant phylum in the intestinal microbiota of laying hens is *Firmicutes*, followed by two smaller phyla, *Proteobacteria* and *Bacteroidota*. In addition, members of the phyla *Actinobacteria* and *Cyanobacteria* can be found in very low abundance. Figure 1 describes the abundance of the phylum found mainly in the excreta of laying hens. There was a reduction in *Actinobacteriota* with a xylanase inclusion of 100 g/MT in the diet (Figure 2). The use of enzymes in the diet promoted an improvement in *Bacteroidota* when compared to the positive and negative control treatment (Figure 2).

The treatment with 50 g/MT of xylanase inclusion increased the abundance of Campylobacterota, and for *Cyanobacterium* (Figure 2) the best results were found with the use of 100 g/MT of xylanase, when compared to the other treatments (*p* < 0.05). *Firmicutes* were reduced with the use of enzymes and the use of XM150 improved Proteobacteria (Figure 2).

## 4. Discussion

This study aimed to evaluate the effects of dietary xylanase addition on performance, egg quality, intestinal health parameters and microbiota in laying hens fed with corn–soybean meal-based diets with reduced ME. The hypothesis tested in this study was that reducing 100 kcal ME/kg of feed during a section of the production phase would negatively affect growth performance, egg quality and intestinal health. It was also hypothesized that increasing levels of xylanase addition could mediate some of the negative effects on growth performance, egg quality and intestinal health with a reduced 100 kcal ME/kg of feed. In this study, xylanase inclusion improved several performance and health parameters in laying hens. Although egg production alone does not consider egg weight, egg mass (g/hen/day) integrates both parameters, offering a more accurate productivity index. It is suggested that xylanase improves nutrient digestibility, enabling better egg mass performance. Since xylanase is able to release energy from nutritional contents that are not digested by the hen’s endogenous enzymes, more dietary energy is available, which can be used for maintenance or egg production, as observed for treatments NC + XM50 and NC + XM100. It is important to note that a lower egg production—and consequently egg mass—was observed for hens fed with the NC + XM150 diet. This could be explained by the inclusion dose being too high for the low feed intake of laying hens, leading to a harsh reduction in digesta viscosity, which could potentially lead to a faster transit time, reducing the availability of nutrient absorption [17].

Despite there being no significant differences in shell strength, this parameter is important for reducing cracked eggs [18,19] and was satisfactory across treatments. Greater eggshell strength was observed for birds fed with NC + XM50 and NC + XM100 and greater eggshell thickness was observed for birds fed with NC + XM100 when compared to the eggs of hens fed with the NC diet. These findings contrast with those observed by [20], who reported a decrease in eggshell thickness with the addition of xylanase in wheat-based diets. The ability of xylanase to release encapsulated nutrients in the digesta [21], such as calcium, making those nutrients available for absorption, may contribute to the observed greater eggshell thickness and consequently eggshell strength, since more calcium would be available for hens.

As for internal egg quality parameters, it was observed that there was a decrease in albumen height for hens fed with either NC + XM50 or NC + XM150 diets. The effect of xylanase decreasing albumen height, and consequently Haugh unit values, was also reported by [22]. As reported by [23], xylanase tends to increase albumen weight and height in diets with adequate phosphorus, while in phosphorus-reduced diets with the inclusion of xylanase, increasing phytase doses can increase albumen weight. The authors hypothesize that the effect of xylanase of increasing the access to dietary nutrients and compounds, such as phosphorus, was not utilized to its full potential, since some of the phosphorus could actually be entrapped in the form of phytate and hens could have received an insufficient dietary phytase inclusion. Nevertheless, it is worth noting that all Haugh unit values amongst treatments were above 72, which is considered excellent egg quality as per USDA standards [16].

Reductions in fecal moisture can be correlated with a lower dirty egg percentage, indicating improved gastrointestinal function and overall hygiene, as supported by [24]. In the present study, no differences in either fecal moisture or dirty egg percentage were observed among treatments (*p* > 0.05). However, a decrease in gut permeability was observed by the dietary inclusion of xylanase (*p* < 0.05). These effects may stem from xylanase promoting beneficial gut microbiota [25,26], reducing pathogenic fermentation and enhancing intestinal integrity [27,28], demonstrating that higher energy levels can serve as a substrate for pathogenic bacteria and impairing bird performance, which may indicate a higher rate of cell renewal and demand for nutrients to maintain the intestinal epithelium. Reducing gut permeability consequently lowers immune activation and energy expenditure, improving performance [29]. Lower gut permeability is beneficial to bird health, since the spaces between intestinal cells adjacent to each other are tighter, allowing for better control of absorption and substance selection. When gut permeability is uncontrolled and increased, there is a loss of intercellular tight junction proteins, leading to gut leakiness and the potential access of harmful substances from the intestinal lumen to the blood [30].

Hematological data reinforced the benefits of lower gut permeability, as fewer monocytes and a balanced lymphocyte profile—suggesting less immune stress [31,32]—were observed. Xylanase also supported enterocyte proliferation and deeper crypts [33], reflecting adaptive responses for enhanced nutrient absorption [34,35]. This response may represent a functional adaptation to the greater availability of substrates for absorption, promoted by the action of xylanase. Similar findings were described by [33], who highlight that the use of exogenous enzymes, such as xylanase inclusion at 100 mg/kg, can stimulate the proliferation of enterocytes as a compensation strategy for reduced digestibility or modulation of the intestinal microbiota. The analysis of intestinal morphology is an essential parameter for evaluating the responses of birds to the diet, as it directly influences the absorption of nutrients and intestinal health. The increased crypt depth may be related to greater cell renewal and the need for greater enterocyte proliferation to maintain the integrity of the intestinal mucosa [33]. This occurs because intestinal crypts are regions responsible for the production of new epithelial cells, which subsequently migrate to the villi to replace senescent cells [36]. The greater depth of the crypts may be a reflection of the greater mitotic activity of intestinal cells, indicating an adaptive effect of the enzyme-enriched diet on the intestinal structure of hens [37].

No differences among dietary treatments were observed concerning butyrate concentrations in the ceca. However, the inclusion of 150 g/MT of xylanase in an energy-reduced diet decreased acetate and propionate cecal concentrations (*p* < 0.05). Since this reduction was not observed for other enzyme-addition treatments, it can be assumed that the decrease in digesta viscosity was excessive due to the use of this higher xylanase dosage, increasing the transit speed in the gut and consequently allowing for a lower fermentation time by microorganisms in the intestinal microenvironment, leading to reduced SCFA concentration.

The composition of the intestinal microbiota of hens is dominated by several bacterial phyla, including *Firmicutes*, *Bacteroidota*, *Proteobacteria*, *Actinobacteriota* and *Campylobacterota*. Changes in the abundance of these phyla can be influenced by factors such as diet, age and management of the hens [38].

Taxonomic analysis showed that fecal bacterial communities were dominated by *Actinobacteriota*, *Bacteroidota*, *Campylobacterota*, *Cyanobacteria*, *Firmicutes* and *Proteobacteria* at the phylum level. The most abundant phylum in the intestinal microbiota of laying hens is *Firmicutes*, followed by two smaller phyla, *Proteobacteria* and *Bacteroidota.* In addition, the phyla *Actinobacteria* and *Cyanobacteria* can be found in very low abundance. There was a reduction in *Actinobacteriota* with a xylanase inclusion of 100 g/MT in the diet. The use of enzymes in the diet promoted an improvement in *Bacteroidota* when compared to the PC and NC treatment. These changes suggest that xylanase can positively modulate the intestinal microbiota, in a way that favors beneficial bacteria to grow while reducing the presence of opportunistic pathogens. *Actinobacteriota* is a phylum of gram-positive bacteria, also known as actinomycetes or actinobacteria. These bacteria have a filamentous, often branched, organization, and despite many members of this phylum being commensal bacteria, a major proportion is also associated with the manifestation of certain diseases in animals. In the context of this study, they represented 4.9% of the phyla found in birds, and their reduction with the inclusion of 100 g/MT of xylanase may indicate a decrease in potential pathogens, contributing to the intestinal health of birds [39]. The greater shift for *Bacteroidota* is particularly relevant, since the bacteria of this phylum are involved in the degradation of complex carbohydrates and in the production of short-chain fatty acids, such as butyrate, which serves as a source of energy for intestinal cells and contributes to the maintenance of the integrity of the intestinal mucosa [40]. In addition, they participate in the metabolism of bile acids and in the transformation of toxic compounds, aiding in their elimination from the body [41]. Despite the reduction in acetate and propionate with the use of 150 g/MT of xylanase, the increased shift of this phylum could have contributed to maintaining the levels of butyrate production in hens fed NC + XM150 diets.

*Firmicutes*, another predominant phylum in the intestinal microbiota of birds, plays a significant role in the relationship between intestinal bacteria and poultry health. Many members of this phylum are able to break down carbohydrates in the intestine that cannot be digested by birds’ endogenous enzymes, such as fiber and resistant starch [42]. When bacteria ferment dietary fiber, they produce metabolites, including volatile fatty acids, such as propionate and butyrate, which can directly act as energy sources for enterocytes, promoting cell growth and differentiation [43], as well as reducing intestinal markers associated with high gut permeability [44]. Despite the reduction in this phylum with xylanase inclusion, it is observed that the microbial shift did not promote negative effects in egg quality, gut permeability or productive performance parameters in laying hens, probably due to the simultaneous increase in *Bacteroidota*.

Altogether, the data indicate that xylanase inclusion promotes a favorable intestinal environment by improving nutrient digestibility, modulating the microbiota, supporting intestinal morphology, and reducing inflammatory and immune responses. These effects synergistically contribute to enhanced egg mass production, egg quality and better general health indicators in laying hens, highlighting xylanase’s role as a promising additive for poultry nutrition.

## 5. Conclusions

The addition of 100 g/ton xylanase to corn–soybean meal and wheat bran-based laying hen diets with a reduction of 100 kcal of ME improved egg production, eggshell strength and intestinal integrity. In addition to hen performance benefits, xylanase addition at 100 g/MT enhanced microbial diversity and richness, promoting a more balanced intestinal ecosystem. This effect is likely due to increased fermentable substrates from arabinoxylan degradation, favoring beneficial phyla such as Bacteroidota. Despite shifts in microbial composition, such as decreases in *firmicutes*, intestinal stability was maintained through functional restructuring of the microbiota. These findings demonstrate that xylanase can act as a modulator of the intestinal microbiota, contributing to the intestinal health and productive performance of birds, especially in diets with higher fiber content, but further studies are necessary in order to better understand the pathways of xylanase on xylan metabolism in laying hens.

## Figures and Tables

**Table 1:** Description of experimental treatments with increasing levels of xylanase in energy-reduced diets for commercial laying hens.

Treatment	Name	Description	XM Activity Per MT
1	PC	Commercial standard diet	-
2	NC	−100 kcal of PC	-
3	NC + XM50	50 g/MT XM added to NC	7500.00
4	NC + XM100	100 g/MT XM added to NC	15,000.00
5	NC + XM150	150 g/MT XM added to NC	22,500.00

**Table 2:** Composition of experimental diets containing increasing levels of xylanase in energy-reduced diets for commercial laying hens.

Parameters	NC ^1^	PC
**Ingredients (% as fed)**		
Ground corn	54.25	54.25
Soybean meal 45%	25.91	25.91
Wheat bran	6.05	6.05
Limestone	9.09	9.09
Dicalcium phosphate	1.57	1.57
Soybean oil	1.00	2.14
Inert (sand) ^2^	1.14	0.00
Vitamin–mineral premix ^3^	0.40	0.40
Salt	0.38	0.38
DL-Methionine	0.21	0.21
**Calculated composition**		
Metabolizable energy (kcal/kg)	2625	2725
Crude protein (%)	17.14	17.14
Calcium (%)	3.90	3.90
Available phosphorus (%)	0.40	0.40
Digestible lysine (%)	0.80	0.80
Digestible sulfur amino acids (%)	0.68	0.68
Digestible threonine (%)	0.58	0.58
Digestible valine (%)	0.72	0.72
Sodium (%)	0.17	0.17
Linoleic acid (%)	1.89	1.89
**Analyzed composition**		
Gross energy (kcal/kg)	3263	3395
Crude protein (%)	17.64	17.87
Moisture (%)	10.83	10.81
Ether extract (crude fat, %)	2.90	3.60
Crude fiber (%)	3.05	5.51
Ash (%)	11.25	8.79
**Sugar profile (%)**		
Xylose	1.23	1.07
Arabinose	1.16	1.07
Mannose	0.13	0.10
Glucose	13.52	12.43
Galactose	1.01	0.88
Fucose and ribose	0.00	0.00

^1^ NC: negative control; PC: positive control. ^2^ The enzyme was added on top of the NC. Soybean oil was replaced by sand to correct the energy content of the diets. ^3^ Vitamin and mineral supplement for laying hens. Guaranteed levels (4 g of product per kg of feed): copper (min) 9 mg/kg; iron (min) 0.05 g/kg; iodine (min) 1 mg/kg; manganese (min) 0.1 g/kg; selenium (min) 0.35 mg/kg; zinc (min) 0.07 g/kg; phytase (min) 300 UI/kg; methionine (min) 0.5 g/kg; folic acid (min) 2 mg/kg; pantothenic acid (min) 15 g/kg; biotin (min) 0.0704 mg/kg; choline (min) 0.27 g/kg; niacin (min) 0.045 g/kg; vitamin A (min) 1000 UI/kg; vitamin B1 (min) 3.3 mg/kg; vitamin B12 (min) 20 mcg/kg; vitamin B2 (min) 8.5 mg/kg; vitamin B6 (min) 50 mg/kg; vitamin D3 (min) 3000 UI/kg; vitamin E (min) 50 UI/kg; vitamin K3 (min) 2.1 mg/kg.

**Table 3:** Effect of increasing levels of xylanase inclusion in energy-reduced diets on the productive performance of commercial laying hens.

Treatment	Egg Production, %	Feed Intake, g	Feed Conversion, kg/Dozen	Egg Mass, g/Hen/Day	Feed Conversion, kg/Egg Mass	Dirty Eggs, %
PC ^1^	89.63 ^ab^	109	1.438	53.56 ^ab^	2.032	4.30
NC	87.83 ^b^	105	1.440	51.32 ^bc^	2.048	3.68
NC + XM50	92.61 ^a^	111	1.423	54.98 ^a^	2.023	3.01
NC + XM100	93.44 ^a^	111	1.421	54.95 ^a^	2.028	2.98
NC + XM150	86.10 ^b^	108	1.503	50.66 ^c^	2.144	3.55
SEM ^2^	0.48	1.11	0.015	0.29	0.022	0.15
P ^3^	<0.0001	0.414	0.538	<0.001	0.488	0.095

^1^ PC: positive control; NC: negative control; NC + XM50: negative control + 50 g/MT xylanase; NC + XM100: negative control + 100 g/MT xylanase; NC + XM150: negative control + 150 g/MT xylanase. ^2^ SEM: standard error of the mean. ^3^ Values followed by different letters in the same column differ significantly by Tukey’s test (*p* < 0.05).

**Table 4:** Effect of increasing levels of xylanase inclusion in energy-reduced diets on egg quality of commercial laying hens.

Treatment ^1^	Egg Weight, g	Albumen Height, mm	Haugh Unit	Shell Strength, Kgf	Shell Thickness, mm
PC	59.82	8.99 ^a^	94.12 ^a^	4.23 ^ab^	0.387 ^ab^
NC	58.42	8.57 ^ab^	92.07 ^ab^	4.01 ^b^	0.379 ^b^
NC + XM50	59.39	8.22 ^b^	89.14 ^b^	4.30 ^a^	0.395 ^ab^
NC + XM100	58.85	8.64 ^ab^	92.44 ^ab^	4.46 ^a^	0.400 ^a^
NC + XM150	58.95	8.41 ^b^	90.67 ^ab^	4.21 ^ab^	0.385 ^ab^
SEM ^2^	0.16	0.06	0.37	0.03	0.002
P ^3^	0.113	0.003	0.002	<0.001	0.041

^1^ PC: positive control NC: negative control; NC + XM50: negative control + 50 g/MT xylanase; NC + XM100: negative control + 100 g/MT xylanase; NC + XM150: negative control + 150 g/MT xylanase. ^2^ SEM: standard error of the mean. ^3^ Values followed by different letters in the same column differ significantly by Tukey’s test (*p* < 0.05).

**Table 5:** Effect of increasing levels of xylanase inclusion in energy-reduced diets on excreta moisture and intestinal barrier integrity of laying hens, assessed by plasma FITC-dextran concentration (ng/mL).

Treatment ^1^	Fecal Moisture, %	Gut Permeability (FIT-C Dextran, ng/mL)
PC	20.10	122.48 ^b^
NC	21.07	87.71 ^ab^
NC + XM50	19.13	76.22 ^a^
NC + XM100	19.39	70.84 ^a^
NC + XM150	18.66	61.01 ^a^
SEM ^2^	0.54	6.67
P ^3^	0.281	0.006

^1^ PC: positive control; NC: negative control; NC + XM50: negative control + 50 g/MT xylanase; NC + XM100: negative control + 100 g/MT xylanase; NC + XM150: negative control + 150 g/MT xylanase. ^2^ SEM: standard error of the mean. ^3^ Values followed by different letters in the same column differ significantly by Tukey’s test (*p* < 0.05).

**Table 6:** Effect of increasing levels of xylanase inclusion in energy-reduced diets on hematological parameters (%).

Treatment ^1^	Heterophils	Lymphocytes	Heterophils/Lymphocytes	Monocytes	Eosinophils	Basophils
PC	31.43	43.14 ^b^	0.88	20.14 ^a^	2.29	3.00
NC	28.43	47.86 ^ab^	0.67	18.86 ^ab^	2.14	2.71
NC + XM50	22.43	55.29 ^ab^	0.40	18.00 ^ab^	2.86	1.43
NC + XM100	24.71	57.86 ^ab^	0.46	12.14 ^ab^	2.71	2.57
NC + XM150	24.17	61.71 ^a^	0.42	9.29 ^b^	2.86	1.43
SEM ^2^	1.717	2.064	0.062	1.316	0.321	0.246
P ^3^	0.507	0.020	0.053	0.025	0.938	0.110

^1^ PC: positive control; NC: negative control; NC + XM50: negative control + 50 g/MT xylanase; NC + XM100: negative control + 100 g/MT xylanase; NC + XM150: negative control + 150 g/MT xylanase. ^2^ SEM: standard error of the mean. ^3^ Values followed by different letters in the same column differ significantly by Tukey’s test (*p* < 0.05).

**Table 7:** Effect of increasing levels of xylanase inclusion in energy-reduced diets on intestinal morphometry (villus height, crypt depth and villus/crypt ratio).

Treatment ^1^	Villus Length, µm	Crypt Depth, µm	Villus Height/Crypt Depth
PC	645.31	128.04 ^b^	5.14
NC	665.29	137.41 ^ab^	4.92
NC + XM50	730.39	164.56 ^a^	4.52
NC + XM100	708.79	162.02 ^ab^	4.40
NC + XM150	664.89	134.55 ^ab^	4.95
SEM ^2^	14.409	4.349	0.118
P ^3^	0.319	0.010	0.230

^1^ PC: positive control; NC: negative control; NC + XM50: negative control + 50 g/MT xylanase; NC + XM100: negative control + 100 g/MT xylanase; NC + XM150: negative control + 150 g/MT xylanase. ^2^ SEM: standard error of the mean. ^3^ Values followed by different letters in the same column differ significantly by Tukey’s test (*p* < 0.05).

**Table 8:** Effect of increasing levels of xylanase inclusion in energy-reduced diets on the concentration of short-chain fatty acids (acetate, propionate and butyrate) in cecal contents.

Treatment ^1^	Acetate, mM/g	Propionate, mM/g	Butyrate, mM/g
PC	111.69 ^a^	49.32 ^a^	21.96
NC	95.67 ^ab^	33.89 ^ab^	21.80
NC + XM50	94.23 ^ab^	30.91 ^ab^	16.25
NC + XM100	88.00 ^ab^	32.62 ^ab^	20.11
NC + XM150	61.19 ^b^	26.55 ^b^	18.05
SEM ^2^	5.28	2.46	1.50
P ^3^	0.034	0.047	0.730

^1^ PC: positive control; NC: negative control; NC + XM50: negative control + 50 g/MT xylanase; NC + XM100: negative control + 100 g/MT xylanase; NC + XM150: negative control + 150 g/MT xylanase. ^2^ SEM: standard error of the mean. ^3^ Values followed by different letters in the same column differ significantly by Tukey’s test (*p* < 0.05).

**Table 9:** Effect of increasing levels of xylanase inclusion in energy-reduced diets on intestinal microbial diversity indexes (Chao1, Shannon and Simpson).

		Index	
Treatment ^1^	Chao1	Shannon	Simpson
PC	662 ^c^	3.58 ^d^	0.928 ^c^
NC	634 ^d^	3.44 ^e^	0.900 ^d^
NC + XM50	711 ^b^	3.84 ^c^	0.936 ^b^
NC + XM100	786 ^a^	4.01 ^b^	0.941 ^b^
NC + XM150	784 ^a^	4.22 ^a^	0.959 ^a^
SEM ^2^	17.5	0.081	0.006
P ^3^	0.005	0.007	0.028

^1^ PC: positive control; NC: negative control; NC + XM50: negative control + 50 g/MT xylanase; NC + XM100: negative control + 100 g/MT xylanase; NC + XM150: negative control + 150 g/MT xylanase. ^2^ Values followed by different letters in the same line differ significantly by Tukey’s test (*p* < 0.05). SEM: standard error of the mean. ^3^ Values followed by different letters in the same column differ significantly by Tukey’s test (*p* < 0.05).

## Data Availability

The data presented in this study are available upon request from the corresponding author.
